# An application of finite element analysis predicts unique temperatures and fates for flatback sea turtle embryos

**DOI:** 10.1242/jeb.250238

**Published:** 2025-12-04

**Authors:** Malindi Gammon, Sabrina Fossette, Ali Karrech, Gavan McGrath, Sami Alkhatib, Nicola J. Mitchell

**Affiliations:** ^1^ School of Biological Sciences, The University of Western Australia, Crawley, 6009 Australia; ^2^ Oceans Institute, The University of Western Australia, Crawley, 6009 Australia; ^3^ The Cawthron Institute, Nelson, 7010 New Zealand; ^4^ Biodiversity and Conservation Science, West Australian Department of Biodiversity Conservation and Attractions, Kensington, 6151 Australia; ^5^ School of Engineering, The University of Western Australia, Crawley, 6009 Australia; ^6^ School of Agriculture and Environment, The University of Western Australia, Crawley, 6009 Australia

**Keywords:** Thermal development, Ecophysiology, Finite element model, Reptile, Abaqus

## Abstract

Incubation temperatures within sea turtle nests are governed by complex abiotic and biotic interactions. Established methods for estimating these temperatures include deploying temperature loggers at standard nest depths or using models to predict sand temperature. While these approaches capture abiotic drivers of incubation temperatures, they often fail to fully account for biotic factors, including the heat produced by the metabolism of embryos, despite its potential impact on hatchling development and mortality.

Here, we applied finite element analysis to predict incubation temperatures for all embryos in five flatback sea turtle (*Natator depressus*) nests. To parameterise the models, we measured clutch size, nest depth, temperatures at several locations around nests, and the physical properties of beach sand, including density and moisture. Next, we simulated within-nest temperatures with an energy balance model that accounted for metabolic heat by applying an hourly heat flux, based on modelled embryonic metabolic rates, to each egg. Each model was validated by comparing predicted temperatures with observed temperatures at the base, centre and top of each clutch. On average, finite element models achieved good accuracy to within 0.4°C of observed data (mean error over the entire incubation period). By incorporating individual clutch size, nest depth and the metabolic contributions of embryos, our approach enhances the realism of temperature estimates for predicting the embryonic development of sea turtles, which is of increasing importance under rapid climate change.

## Teaser

**Summary:** Finite element modelling captures realistic sea turtle nest temperatures by integrating environmental drivers with biological sources of heat.

## INTRODUCTION

Complex abiotic and biotic interactions drive incubation temperatures of egg-laying species with underground nests. These include the transport of heat and water through soil (Bittelli et al., 2015; Campbell, 1985) and the metabolism of developing embryos, which produce heat (i.e. metabolic heat; Deeming, 2004). Consequently, in species with larger egg clutches, no two egg temperatures are identical. Small differences in incubation temperature (as little as ∼0.5°C) can have an appreciable impact on developmental outcomes of reptiles such as sea turtles, which have temperature-dependent sex determination (Bull, 1980). Many reptile species transition from producing all male to all female offspring within a transitional range of temperatures of 1–2°C (Hulin et al., 2009; Mrosovsky and Pieau, 1991), and development rates and hatchling survival are similarly temperature-sensitive (Ackerman, 1996; Angilletta, 2009; Howard et al., 2014). Predicting the primary sex ratios and hatching success of reptile populations under future climates therefore hinges on our ability to realistically model the range of incubation temperatures experienced by each embryo in a nest.

The temperature of the sand adjacent to an egg chamber is a popular proxy for incubation temperature among studies of sea turtles focused on developmental outcomes (e.g. Fuentes et al., 2009; Jensen et al., 2018). However, studies as early as the 1950s found that metabolic heat increases incubation temperatures above that of the surrounding sand in sea turtle nests (Carr and Hirth, 1961; Hendrickson, 1958) and, more recently, in freshwater turtles, which have smaller clutch sizes (Massey et al., 2019). Sea turtle nests typically contain 50–130 eggs, depending on the species and size of the female (Miller, 1996) and each egg generates an increasing amount of metabolic heat as the embryo develops through to hatching. As a result, incubation temperatures may be 2–5°C warmer than the surrounding sand when metabolic rates peak close to hatching (Chen et al., 2010; Gammon et al., 2020; Girondot and Kaska, 2015; Valverde et al., 2010). Therefore, sand temperature proxies for embryonic temperatures are most useful in the first one-third to one-half of development, when metabolic heat is barely detectable. Applying a correction factor to measured sand temperatures, to account for metabolic heat in adjacent nests, remains a valuable and widely used approach for estimating primary sex ratios. Temperature corrections (typically between 0.5 and 1°C) have been applied to the thermosensitive period (TSP) for sex determination, which roughly corresponds to the middle third of development (e.g. Patrício et al., 2017; Tanner et al., 2019; Stubbs et al., 2014). However, accurately capturing metabolic heat is important for understanding spatial variation in nest temperatures, and how this impacts embryonic survival and hatchling traits, considerations that correction factors applied at broader scales overlook.

Modelling metabolic heat is complicated because embryonic metabolic rates vary with incubation temperature and developmental maturity, which is itself governed by incubation temperature (Ackerman, 1980; Reid et al., 2009; Stubbs and Mitchell, 2018). Although complex, these interactions are primarily ruled by physical and chemical processes, making it theoretically possible to predict the heat balance of any embryo within a nest. The temperature sensitivity of biological processes can be described by temperature coefficients (e.g. *Q*_10_; Van't Hoff, 1884) that capture the activation energy of enzyme-catalysed reactions and inform the rate of biological responses to temperature (e.g. the Arrhenius temperature; Arrhenius, 1889). These coefficients can be used to predict reaction rates across biologically meaningful temperatures, including the metabolic rates of sea turtle embryos (Stubbs and Mitchell, 2018). Interactions between neighbouring sources of heat (embryos), and the air and sand surrounding them, drive spatial variation in incubation temperatures within a nest (Kaska et al., 1998a,b; Vázquez Luna et al., 2000). Similarly, nest depth, distance from the high-tide mark, sand moisture and weather patterns also determine how environmental forcing interacts with a nest's energy budget.

Energy balance models, underpinned by finite element analysis, provide a powerful numerical approach to predict the behaviour of complex systems (Reddy, 2005). Finite element analysis works by subdividing a system into individual components or ‘elements’ whose behaviour is averaged (Zienkiewicz et al., 2013). In the case of energy balance models, the temperature of each element at a certain time is then represented by simple equations related to its past temperature and that of its neighbouring elements and any boundary conditions. These equations are then reassembled into a larger system of equations to model the entire system (Zienkiewicz et al., 2013). Finite element analysis is applied in many disciplines, predominantly engineering (e.g. modelling the transfer of heat between engine components; Muhammad and Haruna, 2020), but occasionally also in biology (e.g. modelling the structural complexity of underground ant nests; Qu et al., 2018). A key advantage of this approach, which solves mathematical models in three-dimensions, is the ability to query model predictions at any location across the finite element ‘mesh’.

Here, we apply finite element analysis to predict incubation temperatures for each embryo in a flatback sea turtle (*Natator depressus* Garman 1880) nest and test its ability to reliably reproduce field measurements taken at different locations within an egg clutch. Finally, we demonstrate how predicting temperatures of individual eggs can then be applied to predict incubation duration, sex and hatching success in a typical flatback turtle nest.

## MATERIALS AND METHODS

### Field data

Field data were collected during the 2020/2021 nesting season at a flatback rookery on Thevenard Island in Western Australia. All procedures were approved by the University of Western Australia Animal Ethics Committee (RA/3/100/1673) and the Western Australian Department of Biodiversity, Conservation and Attractions (DBCA; TFA 2019-0092).

#### Nest and sand temperature

Temperature loggers (described below) were deployed in 14 freshly excavated flatback nests over 1 week during December 2020. To achieve this, nightly patrols were conducted for several hours before and after high tide and any nesting turtles were covertly monitored while constructing their egg chambers. One person then observed egg laying to determine when it was complete, at which point the female was immediately lifted off her egg chamber and out of her body pit by 2–3 people and placed approximately 3 m away. This intervention prevented females from filling and packing down sand over the nest, leaving a stable and well-defined egg chamber that could then be instrumented with temperature loggers. Females continued their stereotypical nesting behaviours (e.g. nest covering) in the new location before returning to the water.

All eggs were carefully removed and counted, and the diameters of a subsample of ten eggs from each nest were measured using digital callipers (±0.01 mm; Kinchrome). Nest depth was recorded by laying a wooden rod across the nest opening so that it was flush with the sand surface and measuring the distance from the rod to the nest base (Fig. S1A). Each nest then received three temperature loggers, programmed to record temperature every hour, at the same time of day (i.e. 00:00, 01:00, 02:00 h etc.). To ensure loggers were not dislodged when the nest was later repacked with eggs and sand, or when embryos hatched around 45–50 days later, loggers were fixed to a narrow (12 mm diameter) nylon rod that was inserted vertically into the centre of the empty egg chamber. For each clutch, one logger (HOBO IC-MX2203; ±0.2°C, resolution: 0.01°C) was positioned at the nest base and pre-attached to the rod using electrical tape. Two smaller loggers (iButton DS1921H; ±1°C, resolution: 0.125°C) were then cable-tied to the nylon rod to accommodate varying clutch sizes. The first of these smaller loggers was positioned to record temperatures in the middle of the clutch, and so it was attached to the rod after half the eggs had been carefully replaced within the egg chamber. The remaining eggs were then added to the nest, and the second iButton attached to the rod so that it was positioned among the top layer of eggs. Finally, the egg chamber was loosely filled with damp sand, and the body pit filled and covered with drier sand to closely resemble the natural nest covering process. Together, all procedures were completed in 1–1.5 h, well below the maximum period of 12 h that sea turtle eggs can be handled without inducing mortality (Limpus et al., 1979; Parmenter, 1980). Temperature loggers (iButton DS1921H) were also deployed in the sand directly above the egg chamber, 10 cm below the sand surface and 100 cm adjacent to the egg chamber, at the same depth as the logger positioned in the clutch centre (Fig. S1). Nest locations were recorded using a differential GPS (EMLID Reach RS+RTK Global Navigation Satellite System) and left to incubate naturally.

Logger uncertainty over 24 h with an hourly sampling rate (i.e. uncertainty of the average temperature) was calculated using the ‘uncertainty.datalogger’ function of the R package ‘embryogrowth’ (https://CRAN.R-project.org/package=embryogrowth; Girondot, et al., 2018) and found to be 0.45°C and 0.09°C for the small (iButtons) and large (HOBO) loggers, respectively. Previous work has shown similar temperature loggers can reliably characterise the thermal conditions on nesting beaches to model the embryonic development of sea turtles (Staines et al., 2022).

#### Sand moisture and density

Marked nests were spread across ∼0.6 km of coastline and located at similar distances above the high tide. Sand properties were calculated for four representative nests, which were evenly spaced across the study area. Immediately adjacent to each nest, three replicate sand cores (length: 6.5 cm, volume: 310 cm^3^) were collected at three depths (0–6.5 cm, 30–36.5 cm and 50–56.5 cm). These resulting ‘beach sand’ samples were immediately weighed (wet weight±0.01 g, QHW-3-plus MiNi, WEIGH Pty Ltd), then oven dried for 6 h at 105°C before re-weighing (dry weight). Dry bulk density (kg^−1^ m^3^) and moisture (percentage of mass) were calculated using these wet and dry weights (Supplementary Materials and Methods Eqns S1 and S2, respectively, Table S1). A soil moisture probe (EnviroPro EP100GL-04) was deployed immediately adjacent to each nest, and at the same depth as the eggs (20–50 cm vertical profile below the sand surface).

**Fig. 1.**
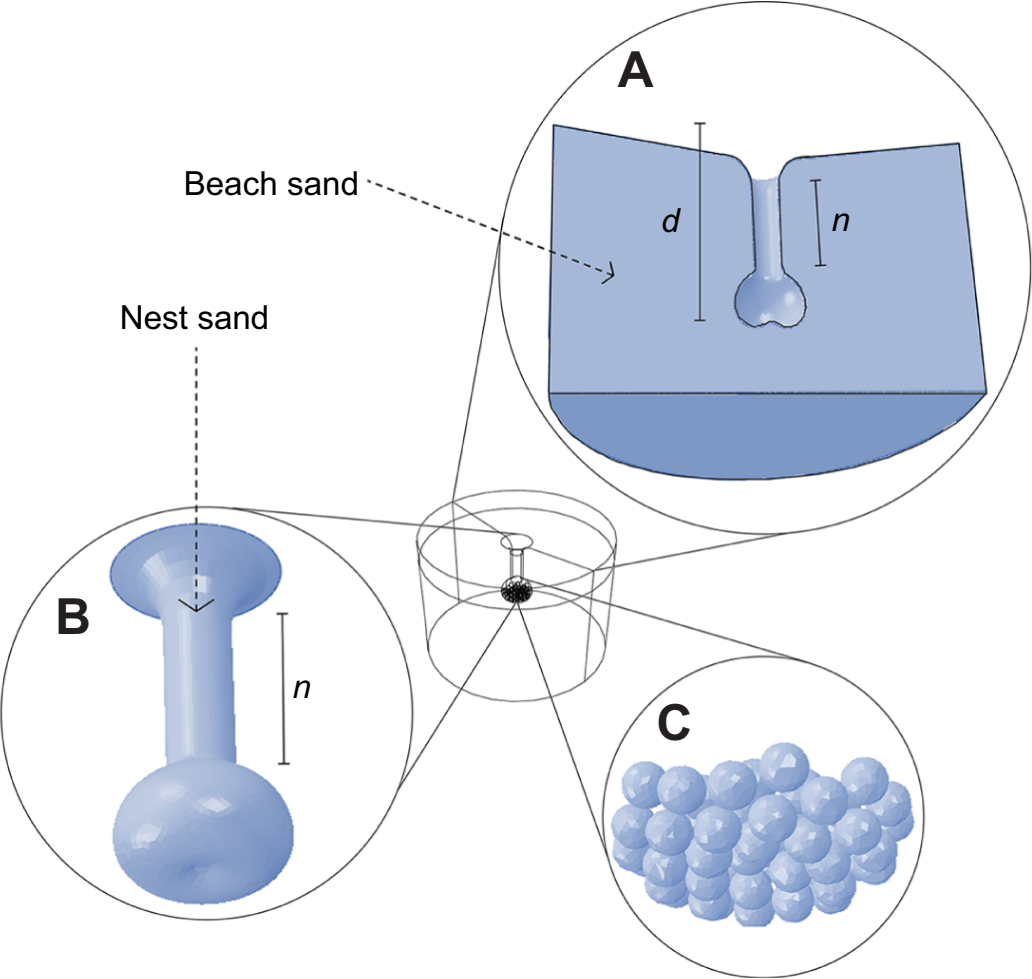
**Subdomains of the finite element model of each flatback turtle nest.** Subdomains represented the surrounding beach (A) the egg chamber (B) and the clutch of eggs (C). The depth of each nest (*d*) was adjusted to reflect observed nest depths by scaling the length of the nest neck (*n*). The example shown has a nest depth of 60 cm and clutch of 51 eggs.

The density of sand in an egg chamber differs from the density of surrounding beach sand because of the way a female turtle excavates and repacks sand during the nesting process. The density of this sand (hereafter referred to as ‘nest sand’, Fig. 1) was estimated in two steps. First, we divided the total mass of sand returned to a nest by the volume of unoccupied space within the egg chamber. Sand mass was calculated by multiplying the density of beach sand by the volume of sand returned to the nest, which was estimated in the field using a 6 liter bucket. The volume of unoccupied space in the nest was calculated by subtracting the total clutch volume from the egg chamber volume. Second, an estimate of the wet and dry density of nest sand was then derived by using wet and dry densities of beach sand in the mass calculations. This process was completed for a single nest, and the estimated sand densities thereafter used for all nests in the study (Table S1).

#### Nest excavations

Once all nests had hatched (∼55 days after the last nest was constructed), they were relocated using the GPS and excavated. Nest contents were analysed to assess hatching success (i.e. the number of hatched eggs as a proportion of the total clutch size at oviposition; Miller, 1999); hatched eggs were identified from shell parchments and only counted as ‘hatched’ if they consisted of more than half of an entire eggshell (Miller, 1999). All unhatched eggs were opened and categorised as either: (1) undeveloped (i.e. no visible signs of an embryo), (2) pre-term embryo (i.e. an obvious embryo which was not yet full-term), or (3) full-term embryo (i.e. an apparently full-term embryo with a very small amount of external yolk) (DBCA, 2018). The metabolic heat produced within each nest was calculated for each hour of incubation using the temperature difference between the central nest logger and the logger positioned at the same depth in adjacent sand, as per standard practice in sea turtle studies (Gammon et al., 2020). Hatching times were estimated based on the temperature profiles of each nest, with hatching assumed to have started when nest temperatures declined (independent of sand temperatures), indicating the beginning of nest emergence.

All statistical analyses were conducted using R software (version 3.6.0) and results presented as means±s.e.m. (unless otherwise stated).

### Building the finite element models

Of the 14 nests marked and excavated, five were suitable candidates for a finite element model. Most nests were unsuitable because temperature loggers deployed directly above the egg chamber (and 10 cm below the sand surface) were either lost, or were no longer at their original depth when retrieved. Depth changes were detected by plotting sand temperatures to reveal any abrupt changes in diel fluctuations, which indicated that sand had either accumulated or been removed from above the egg chamber because of a disturbance (typically another nesting female, or sand shifting in strong winds). We note that abrupt changes in nest temperature also occur following heavy rainfall (Staines et al., 2020), but the changes in diel temperature fluctuations we detected did not correspond with rainfall events (Fig. S2), supporting the view that the volume of sand above the nest had changed instead. Hence each of the five nests we chose for a finite element model each had complete temperature data and a stable sand volume above the egg chamber throughout the incubation period.

Finite element models for each of the five nests were built in Abaqus (2017), a general-purpose software package that uses the finite element method (www.3ds.com). Before heat transfer could be reliably modelled, model parameters describing nest geometry, the thermal behaviour of nest materials, and initial and boundary conditions (which are known conditions – in this case sand temperature) had to be specified. Broadly, each model had three subdomains, representing the undisturbed beach sand surrounding egg-chambers, the egg chamber, and eggs (Fig. 1). Consistent parameters across models included the shape and volume of egg chambers, and the material properties of model domains. Parameters adjusted to reflect field observations for each selected nest included nest depth, the number of eggs (total clutch size at oviposition; Table 1), and the heat flux of individual embryos. Additionally, based on the estimated mortality stage of unhatched eggs, both the number of embryos contributing heat and heat flux durations were adjusted accordingly to assess whether model accuracy could be further improved (see ‘Boundary conditions and metabolic heat fluxes’ for full details). For all nests, results from models that applied heat fluxes to all eggs (regardless of mortality) were compared with models that accounted for embryo mortality.

**Table 1.** Nest-specific characteristics for each model

Model/nest	Nest depth (cm)	Length of nest neck (cm)	Clutch size (no. eggs)
1	42	11	38
2	73	42	43
3	60	29	51
4	61	30	59
5	64	33	61

Specific parameters relating to the number of eggs that received a heat flux are provided in Table S2.

#### Model

For all models, the geometry of the subdomain representing beach sand (i.e. the undisturbed sand surrounding egg chambers) was defined by a cylinder of 2 m diameter and 1.5 m depth, and the geometry of the egg chamber was defined using average dimensions for flatback turtle nests from the literature (Fig. S1; Koch et al., 2007). Egg geometry was captured by assuming each egg was a sphere of 46.7 mm diameter (based on the average diameter of eggs measured in the field) and clutch size was adjusted to reflect the observed number of eggs in each nest at oviposition, ranging from 38 to 61 eggs (Table 1). Nest depth was adjusted to match field observations for each nest, ranging from 42 to 73 cm below the sand surface (Table 1). To reflect the random placement of eggs within an egg chamber, eggs falling into the nest were simulated using a ‘motion analysis’ with enabled gravity in SolidWorks (www.solidworks.com). Domain geometry was also created in SolidWorks.

#### Thermal properties of subdomain materials

Each subdomain consisted of different materials, primarily eggs and either beach or nest sand. Thermal properties – density, thermal conductivity and specific heat capacity – were derived for beach and nest sand using the core components of these materials, which include sand, air and water. For eggs, we made the simplifying assumption they shared the same thermal properties as water. The final thermal properties applied across all models for each subdomain are given in Table 2, and the process and fuller justification for these calculations are provided below.

**Table 2.** The thermal properties of subdomain materials

Properties	Materials		
	Beach sand	Nest-chamber sand	Eggs
Density (kg m^−3^)	1282.2	1026.6	1000
Thermal conductivity (W m^−1^ C^−1^)	2.2	1.7	0.6
Specific heat capacity (J kg^−1^ C^−1^)	829.9	661.3	4177.6

The density of beach sand was calculated by pooling data for sand cores taken from 30–36.5 cm and 50–56.5 cm, because the physical properties of sand did not vary greatly over this depth range (Table S1). Using this approach, the density of beach sand was 1282.2±73.9 kg^−1^ m^3^ and sand moisture was 4.0±0.8%. The density of nest sand was 1025.6 kg^−1^ m^3^ (calculated from one nest) and sand moisture was assumed to be the same as beach sand (4.0±0.8%).

The principal of homogenisation was used to estimate the thermal conductivity and specific heat capacity of beach and nest sand. Specifically, nest sand is a mixture of sand (we assumed that sand was quartzite), water and air, and we predicted the thermal properties of nest sand using the thermal properties of these materials (Table S3; Karrech et al., 2021). Thermal conductivity was calculated using Mori–Tanaka's approach to homogenisation (Mori and Tanaka, 1973; Supplementary Materials and Methods Eqn S3), and the volume fractions of each material (Table S4). Using this approach, the thermal conductivity for beach sand and nest sand was 2.2 W m^−1^ °C^−1^ and 1.7 W m^−1^ °C^−1^, respectively. Volumetric heat capacity was calculated using the rule of mixtures (Laloui and Loria, 2020; Supplementary Materials and Methods Eqn S4) and was 829.9 J kg^−1^ °C^−1^ and 661.3 J kg^−1^ °C^−1^ for beach sand and nest sand, respectively.

We assumed that freshly deposited eggs retained the thermal properties of water throughout development until hatching (Table S3). This was a reasonable assumption based on the high water content of flatback eggs and hatchlings (79% and 77% of the mass, respectively; Hewavisenthi and Parmenter, 2002) and similar densities to water (within 6%) at both the egg stage in other reptiles (Webb et al., 1987) and adult stage in a different sea turtle species (leatherback, *Dermochelys coriacea;* Fossette et al., 2010) (Table S5). Given that water has a relatively low thermal conductivity compared with the biological materials that make up the remaining ∼20% of sea turtle embryos, it is likely we slightly underpredicted the thermal conductivity of eggs.

#### Boundary conditions and metabolic heat fluxes

Hourly sand temperatures measured adjacent to each nest (at the same depth as the clutch centre, ranging from 34–60 cm) and directly above each nest (10 cm below the sand surface) were used as time-varying temperature boundary conditions for each model. These boundary conditions accounted for heat fluxes outside of each nest, including those from solar radiation and rainfall. The metabolic heat generated by developing embryos was captured by applying an hourly heat flux to the central node of all eggs, which were first assumed to be viable. A second model, adjusted to reflect mortality, had heat fluxes applied as follows: (1) embryos that hatched or pipped received a heat flux for their entire incubation period; (2) embryos that reached ‘full term’ before dying also received a heat flux for their entire incubation period; (3) eggs that died and reached ‘pre-term’ had their heat flux terminated at two-thirds of development; and (4) eggs that showed no signs of development did not receive a heat flux. This adjusted model was constructed for all five nests, so that the proportion of eggs in each category matched field observations (Table S2), with clutch positions of eggs in each of the four categories assigned randomly.

Hourly heat flux values for each nest were calculated based on the average hourly incubation temperatures recorded at three points within the clutch, using the following three steps.

First, we calculated the cumulative amount of embryonic development completed (with 100% denoting hatching stage) on an hourly time step, as a function of observed nest temperatures, using a non-linear development rate function generated in a previous study of the same flatback turtle population (Supplementary Materials and Methods Eqn S5).

Second, we calculated the metabolic heat (mW per embryo) each embryo was expected to produce as a function of its maturity (0–100%), using a previously established equation fitted to laboratory data from flatback embryos incubated at 29.5°C (Eqn S6). In brief, metabolic heat was estimated by converting embryonic metabolic rates (*V̇*_CO_2__ and *V̇*_O_2__) to their energy equivalence (Gammon et al., 2021).

Thirdly, heat fluxes were adjusted from 29.5°C to those appropriate for each hourly temperature using *Q*_10_ temperature coefficients (Eqns S7 and S8), which were based on the expected development rates at each temperature (Eqn S5). An example of the temperature- and time-dependent heat flux calculated for nest 3 is shown in Fig. S3.

#### Governing equations

The following equations were applied across model elements in Abaqus to capture the relationship between the thermal behaviour of each domain, and the biological source of heat from each embryo:
(1)

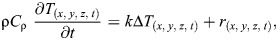
where, ρ is density (kg^−1^ m^3^), *C*_ρ_ is specific heat capacity (J kg^−1^ °C^−1^), ∂T/∂t is rate of change of temperature (°C), *k* is thermal conductivity (W m^−1^ C^−1^), Δ*T is* Laplacian of temperature (°C m^−2^); *r is* metabolic heat generation (W) (coincides with the metabolic heat generation where there is an egg and takes zero elsewhere), *x*, *y*, *z* are space coordinates and *t* is time.

Heat is transferred through the sand, between the sand and eggs, and between adjacent eggs, via conduction. The flux of heat conduction was calculated by Fourier's Law:
(2)

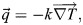
where, *q* is heat flux (W m^−2^); *k* is material conductivity (W m^−1 ^°C^−1^) and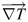 is the temperature gradient (°C m^−1^).

#### Model performance

We tested the ability of the model to accurately replicate incubation temperatures by comparing observed temperatures within each clutch (i.e. from temperature loggers deployed in model nests) to modelled temperatures at similar locations. Modelled temperatures were extracted by selecting the central node of the egg closest to the three locations where we had observed data (i.e. at the base, centre and top of a clutch). Data were extracted at eight-hourly time steps that exactly matched those of the observed data (i.e. 00:00, 08:00, 16:00 h). The relationships between observed data and model predictions were compared using the root mean squared error (RMSE), the coefficient of determination (*R*^2^), the mean error and mean absolute error, using the ‘caret’ R package (https://CRAN.R-project.org/package=caret; Kuhn, 2008).

### Predicted developmental outcomes

#### Integration of modelled temperatures with a physiological model

With the goal of illustrating a framework to predict individualised developmental outcomes for each embryo, we integrated results from the finite element model (hourly incubation temperatures) with an existing model of embryonic development in reptiles with temperature-dependent sex determination (DEVOUR; Bentley, 2018; Carter, 2015). The nest with a depth and clutch size that best reflected a typical flatback nest was chosen for this exercise and an unadjusted model (i.e. one with heat fluxes applied to all eggs) was used (Table 1, nest 3). To extract values that represented the temperature of each of the 51 embryos in nest 3, one observation node from the finite element mesh was selected at the centre of each egg over 55 days (the maximum modelled incubation duration for an egg in this nest).

Each set of hourly incubation temperatures were used as separate inputs for the R function DEVOUR. This function uses species-specific parameters to define the relationship between temperature and development rate, as well as temperature and hatchling sex, to calculate the expected incubation duration and sex of hatchings (see Gammon et al., 2024 for a full description of this model and the parameters used). Hatching success was estimated following Laloë et al. (2017) who assembled a database on the relationship between average incubation temperature and hatchling mortality for three sea turtle species. This relationship is modelled by a logistic regression and identifies 32.7°C as a critical inflection point above which hatchling mortality significantly increases in sea turtles. The three developmental outcomes (incubation time, sex and hatching success) were calculated for all 51 embryos and then averaged and compared with a single value estimated by the DEVOUR function based on the sand temperature 1 m from the egg chamber (i.e. the observed boundary temperature). This comparison aimed to assess whether individualised developmental outcomes based on finite element analysis were substantively different to those of a generalised prediction based on a single incubation temperature.

#### Comparison between methods to estimate sex ratios

For all five nests, we estimated sex ratios using established methods to estimate incubation temperatures, including: (1) the average sand temperature adjacent to each nest (and at the same depth as the clutch centre), and (2) adjusted sand temperatures +0.5°C, +1.0°C and +1.5°C above adjacent sand temperatures. These established methods were compared with temperatures modelled using finite element analysis. In all cases, average temperatures during the middle third of development, corresponding to the TSP were calculated and then converted to a sex ratio based on the temperature-dependent sex determination reaction norm for this genetic stock of flatback turtles (Gammon et al., 2021), implemented via the DEVOUR function.

## RESULTS

### Field data

All marked nests (*n=*14) were successfully located and excavated. Incubation temperatures of central eggs exceeded adjacent sand temperatures by 0.7°C and 2.7°C during the second and third trimesters, respectively (Table 3), indicating the magnitude of metabolic heating. No metabolic heat was detected during the first trimester. Average incubation temperatures at the clutch base were almost 1°C cooler than eggs at the clutch surface (Table 3). Average hatching success was low (42%) across all nests, including the five nests selected for subsequent finite element modelling (37%) (Table 3). However, very few eggs showed no signs of embryonic development (on average just 6% and 4% for all nests and modelled nests, respectively) and, among eggs which did not hatch in modelled nests, 30% were classified as full-term embryos (Table 3). Hourly readings of sand moisture adjacent to four nests showed that the moisture content of sand remained stable throughout the season, indicating it was appropriate to assume that sand moisture also remained stable in subsequent finite element models (Fig. S2).

**Table 3.** Observed data for key parameters of flatback turtle nests* on Thevenard Island, including the proportion of eggs in clutches which hatched (i.e. hatching success), were unviable (i.e. no development) or failed to hatch but developed to pre-term or full-term

Parameter	Mean±s.e.m.			*n*
Clutch size (*n* eggs)	47.8±2.6 (50.4±4.0)			14 (5)
Hatching success (%)^‡^	42.0±8.0 (37.4±11.8)			14 (5)
No development (%)	6.5±1.1 (4.7±1.0)			14 (5)
Dead pre-term embryos (%)	32.7±5.3 (31.1±4.8)			14 (5)
Dead full-term embryos (%)	17.5±6.6 (25.7±11.5)			14 (5)
Nest depth (cm)	59.3±9.9 (60.0±4.5)			14 (5)
Incubation temperatures at different clutch locations (°C)^§^	Base	Centre	Surface	14 (5)
	31.8±0.0 (32.2±0.5)	32.5±0.0 (32.9±0.6)	32.7±0.0 (33.1±0.5)	
Incubation temperatures per trimester (°C)^¶^	First	Second	Third	14 (5)
	30.1±0.2 (30.5±0.1)	31.9±0.3 (32.2±0.3)	35.6±0.2 (36.0±0.1)	
Metabolic heat per trimester (°C)**	First	Second	Third	12 (5)
	−0.1±0.0 (0.2±0.3)	0.7±0.0 (1.0±0.5)	2.7±0.0 (3.2±0.6)	

*Parameters for the subset of five nests modelled using finite element method are provided in parentheses (also see Table S2).

^‡^Hatching success in this study was comparable to unmanipulated nests at the Thevenard Island rookery during the same season (46±4%; D.B.C.A., unpublished data).

^§^Average temperature between oviposition to hatching at three positions in each clutch.

^¶^Average temperatures at the clutch centre, for each trimester of incubation.

**Measured as the difference between incubation temperatures at the centre of each clutch, and at the same depth 1 m adjacent to each clutch. Data are reported for each trimester of incubation.

### Model performance

**Fig. 2.**
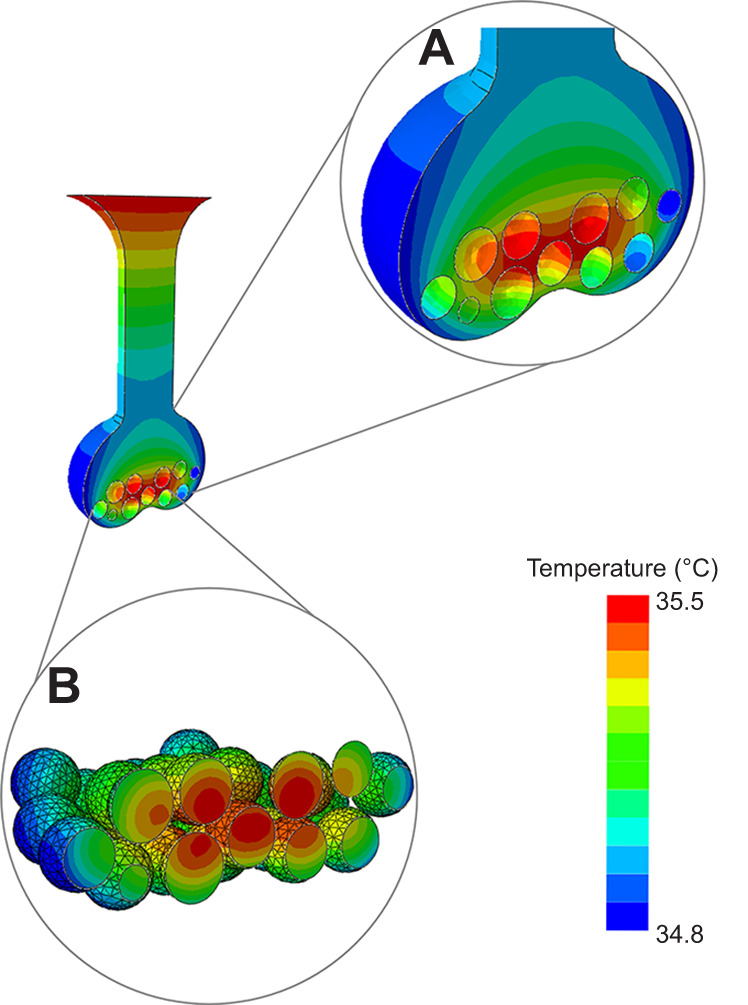
**An example snapshot of the modelled temperature of the sand and eggs just prior to hatching (∼day 48) for nest 3.** Sand temperature (A) declines with distance from the sand surface while embryos at the centre of the clutch (B) experience warmer temperatures than those at the sides and base of the clutch. This snapshot represents midday, when solar radiation is at its peak, resulting in the high temperatures at the sand surface. In contrast a snapshot at midnight (not shown) revealed that temperatures at the sand surface fall below that of the eggs. The stippling on the outside of the eggs in B shows the mesh applied for finite element analysis (the mesh is not shown in other figures).

**Fig. 3.**
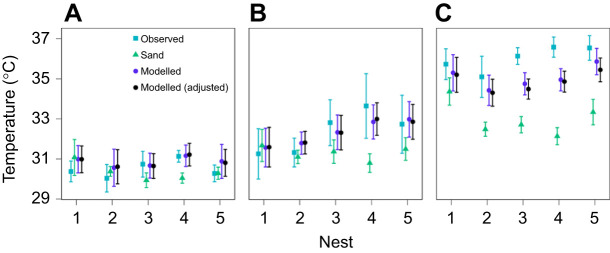
**Comparison of methods to predict the incubation temperature of central eggs in a clutch of sea turtle eggs.** Data (means±s.d.) are presented for five nests during each trimester of incubation: (A) first, (B) second and (C) third trimester. Data shown as either measured incubation temperatures at the clutch centre (squares), the temperature of sand 1 m adjacent to the egg chamber (triangles) or temperatures based on energy balance models underpinned by finite element analysis (circles). These data are also presented as time series in Fig. S4.

Finite element models generated temperature variation within the egg chamber and between individual eggs (see Fig. 2 for an example of a nest close to hatching stage). Modelled incubation temperatures (*n=*5 nests) were a good match to observed incubation temperatures at each location within the clutch (*R*^2^>0.9) and were generally within 0.4°C of observed values (Fig. 3, Table 4). On average, models that were not adjusted for mortality performed slightly better than adjusted models, although this difference was marginal (Table 4, Fig. 3).

**Table 4.** Model performance* at different locations within each clutch

Clutch location	Metric	Nest^‡^					Average^§^
		1	2	3	4	5	
Base	RMSE	0.64	0.73	0.48	0.90	1.05	0.76
	*R* ^2^	0.99	0.96	0.98	0.91	0.87	0.94
	MAE	0.54	0.66	0.43	0.76	0.92	0.66
	ME	0.51	0.43	0.11	0.62	0.52	0.44
Centre	RMSE	0.54	0.66	0.89	1.20	0.99	0.86
	*R* ^2^	0.98	0.96	0.98	0.92	0.88	0.94
	MAE	0.49	0.59	0.69	0.88	0.84	0.70
	ME	0.17	0.09	−0.62	0.67	0.00	0.06
Centre (adjusted)^¶^	RMSE	0.57	0.74	1.03	1.25	1.08	0.93
	*R* ^2^	0.98	0.96	0.98	0.83	0.92	0.93
	MAE	0.52	0.66	0.78	1.06	0.88	0.78
	ME	0.14	0.09	−0.71	−0.20	0.04	−0.13
Surface	RMSE	0.22	0.62	0.62	1.03	0.80	0.66
	*R* ^2^	0.99	0.95	0.99	0.88	0.91	0.94
	MAE	0.17	0.53	0.53	0.83	0.56	0.52
	ME	0.05	0.01	−0.50	−0.34	−0.17	−0.19

*Model performance was examined by calculating root mean square error (RMSE), the coefficient of determination between observed and predicted values (*R*^2^), the mean error (ME) and mean absolute error (MAE), for each location within the clutch. A lower RMSE indicates closer agreement between observed and predicted values, and a positive ME indicates that modelled temperatures were higher than observed, and vice versa.

^‡^Numbers refer to the nests in Table 1, and the corresponding nest-specific characteristics of the model.

^§^Average differences between observed and modelled data for all nests.

^¶^Central temperatures of models adjusted to account for observed embryo mortality.

During the second and third trimesters when metabolic heat was detected (Table 3), the finite element model was a more accurate prediction of incubation temperature compared with sand temperature. On average, sand temperatures during the middle trimester were 1.4±0.1°C lower than measured nest temperatures, whereas modelled temperatures were 0.2±0.0°C lower. Sand temperatures during the last trimester were 2.8±0.1°C lower than nest temperatures, whereas modelled temperatures were 0.7±0.0°C lower. Related to this result, we found that modelled temperatures captured some of the observed temperature variation within each clutch, but modelled variation was consistently lower than the observed values, particularly for larger clutches (Table S6).

### Predicted developmental outcomes

#### Integration of modelled temperatures with a physiological model

**Fig. 4.**
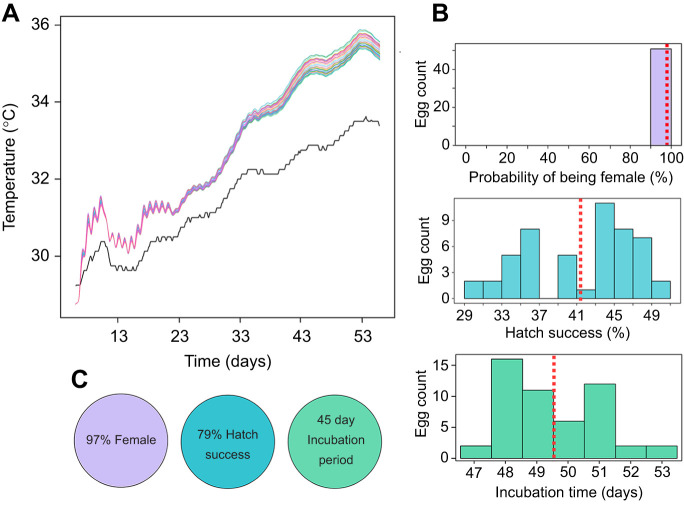
**Modelled incubation temperature of nest 3, generated using the finite element method and the range of developmental outcomes calculated based on these temperatures, including sex, hatching success and incubation duration.** (A) Model temperature of each of the 51 eggs in nest 3, generated using the finite element method (coloured lines). The solid black line shows the observed sand temperature 1 m adjacent to the clutch. The gradual increase in sand temperature over time is consistent with seasonal warming driven by increasing solar radiation and air temperatures. This pattern is typical for early-season nests at summer nesting beaches at high latitudes. (B) Range of developmental outcomes including sex, hatching success and incubation duration, calculated based on the temperatures from A. Dashed red lines show the average of these values. (C) Corresponding developmental outcomes predicted based on this single temperature. The observed hatching success of nest 3 was 67%.

Average modelled incubation temperatures for each of the 51 embryos in nest 3 varied by 0.5°C between the coolest and warmest regions of the nest during the final third of incubation (35.0±0.2–35.5±0.2°C). As expected, this variation was greatest towards the end of incubation when central eggs were the warmest (Fig. 2). Although small, this variation drove subtle differences in modelled developmental outcomes for individual embryos. For example, the average predicted incubation duration across 51 embryos was 49.5±1.5 days, but varied by 6 days between individuals (47–53 days) (Fig. 4). All embryos had a 99% likelihood of developing to be female, indicating complete feminisation of the clutch, but there were no empirical data to confirm this. Predicted average hatching success was 41.5±0.8% (range: 29.6–50.2%; Fig. 4) which was lower than this nest's observed hatching success of 67%. However, 29% of eggs did not hatch but reached a pre-term stage of development, whereas just 4% showed no signs of development.

The average (observed) temperature of the sand adjacent to this nest was 1.9°C cooler (31.5±0.1°C) than the average (modelled) incubation temperature of the 51 embryos (33.4±0.2°C). Consequently, this led to differences in the predicted developmental outcomes: a shorter incubation period (45 days), higher hatching success (79%) and 3.4% of hatchlings being male (Fig. 4).

#### Comparison between methods to estimate sex ratios

Different methods were used to estimate the average incubation tempertaure during the TSP, including: observed sand temperature, corrected sand temperature, and modelled temperature derived from finite element analysis. Among them, sex ratios derived from the modelled temperatures showed the closest agreement with those calculated from observed incubation tempertures (Table 5). However, differences between the modelled outcomes were marginal. With the exception of uncorrected sand temperatures, most model estimates, regardless of the method used to estimate average temperature, were within 3% of those derived from observed temperatures at the clutch centre.

**Table 5.** Estimated sex ratios* of the five modelled nests based on average temperatures (±s.e.m.) during the thermosensitive period (TSP)

Metric	Nest				
	1	2	3	4	5
Observed (°C)	31.3±0.2	31.3±0.1	32.8±0.2	33.6±0.2	32.7±0.1
Sand (°C)	31.7±0.2	31.1±0.0	31.4±0.1	30.8±0.1	31.5±0.1
Modelled (°C)^‡^	31.6±0.1	31.8±0.1	32.3±0.1	32.9±0.1	32.9±0.1
% Female (observed)	92.7	93.3	99.4	99.8	99.3
% Female (sand)	96.2	90.6	93.8	85.0	95.0
% Female (modelled)^‡^	95.6	96.9	98.6	99.5	99.5
% Female (sand+0.5)	98.3	95.7	97.2	93.1	97.7
% Female (sand+1.0)	99.2	98.0	98.7	96.9	99.0
% Female (sand+1.5)	99.6	99.1	99.4	98.6	99.5

Temperatures were either recorded at the clutch centre (i.e. observed), adjacent to nests (i.e. sand) or modelled using finite element analysis. Sex ratios based on increasing sand temperatures by 0.5, 1.0 and 1.5°C above observed values are also shown. Sex ratios that differed substantially from those based on observed incubation temperatures (considered the reference) are underlined.

*The temperature-dependent sex determination (TSD) reaction norm for this flatback turtle stock has parameters *P* at 30.3 and *S* at −0.01 (Hill model), resulting in a pivotal temperature of 30.3°C and a transitional range of temperatures (TRTs) where both sexes are produced between 29.4 and 31.2°C (Gammon et al., 2021).

^‡^Models not adjusted to reflect embryo mortality.

## DISCUSSION

Sand temperatures are widely used by sea turtle researchers to predict embryonic development. Many studies either: (1) assume sand temperatures are broadly representative of nest temperatures, or (2) apply a correction factor to account for the biological sources of heat modelled here. The absence of this correction factor makes sand temperatures several steps removed from the actual temperatures experienced by embryos (Wyneken and Lolavar, 2015). Even when applied, correction factors are often used at the scale of the whole clutch or beach and ‘correct’ for discrete periods of development (i.e. when sex determination occurs). While preliminary, our novel application of an energy balance model, underpinned by finite element analysis, improves our understanding of the dynamics of the nest microclimate. Because the method we describe accounts for both external drivers of incubation temperatures (i.e. solar radiation and rainfall) and biological factors such as metabolic heat, egg chamber morphology and the spatial arrangement of eggs, it builds useful realism into models of incubation temperature.

### Sand temperature is a useful metric for broadly assessing sex determination

During the second and third trimesters, finite element model temperatures were within 0.6°C and 0.9°C degrees of observations, respectively. Meanwhile, observed nest temperatures gradually diverged from those of the adjacent sand by 1.6°C during the second trimester and 2.8°C during the third trimester. These differences are attributed to metabolic heating given that late-stage embryonic development was achieved in all nests, and are consistent with observations of other sea turtle species, where metabolic heating peaked a few days to several hours before hatching (Chan and Liew, 1995; Howard et al., 2014; Neville et al., 1998; Sari and Kaska, 2016). Because many studies that predict primary sex ratios from sand temperatures apply a temperature correction during the TSP of between 0.5–1.0°C (e.g. Esteban et al., 2016; Godley et al., 2002; Patrício et al., 2017; Tanner et al., 2019), our results broadly support this practice, particularly when corrected temperatures are well outside the transitional range producing mixed sexes (Table 5). However, metabolic rates scale non-linearly with developmental stage and temperature, and a fixed correction applied to the TSP will not capture peak metabolic rates that occur afterward, which are consequential for predicting embryonic mortality.

### The impact of metabolic heat on embryonic development

Both our observations and finite element analysis showed that embryos at the top of the clutch generally experienced the warmest temperatures until the final third of incubation, when metabolic heating caused the temperatures of central embryos to resemble those at the top. This phenomenon has also been shown empirically for green and loggerhead sea turtles (Kaska et al., 1998a,b; although see Booth and Astill, 2001a and Booth and Freeman, 2006) and highlights the need to capture biological sources of heat when aiming to predict developmental outcomes in reptiles with large clutch sizes. For metabolic heat to have a consequential impact on embryonic sex determination, sand temperatures during the TSP must be sufficiently cool to produce some male hatchlings. In such cases, biological sources of heat may be sufficient to increase the number of female hatchlings (Broderick et al., 2001). While male-producing sand temperatures are increasingly rare owing to rapid climate change (reviewed by Patrício et al., 2021), examples of rookeries that produce balanced or male-dominated primary sex ratios do exist (Laloë et al., 2020; Patrício et al., 2017; Stubbs et al., 2014). At warmer female-producing beaches, metabolic heat is inconsequential for sex ratio modelling, but consequential for predicting incubation times and mortality, particularly when ambient sand temperatures are near lethal thermal limits. Accounting for biological sources of heat should allow for more refined estimates of primary sex ratios and hatching success, each of which are key life history parameters when forecasting a population's extinction risk (Mitchell et al., 2010; Maurer et al., 2021).

By analysing heat transfer across all components of a sea turtle nest (including eggs, sand and air pockets) and coupling these temperature predictions to a model of embryonic development, we predicted variation in developmental outcomes. This demonstration, based on a single but broadly representative flatback nest in terms of its depth and clutch size, showed that incubation duration is expected to vary up to 6 days (47–53 days). Such variation is found in natural nests and is reflected in the asynchrony of hatchling emergence (Glen et al., 2003; Houghton and Hays, 2001; Koch et al., 2008). When developmental predictions were based on the lower temperature of the adjacent sand, the estimated incubation duration fell to 45 days (i.e. development rates were faster). While counterintuitive, this difference is because incubation temperatures predicted by the finite element model exceeded the temperature where modelled development rates peak for flatback sea turtles (between 33 and 34°C; Bentley et al., 2020b; Gammon et al., 2021; Stubbs and Mitchell, 2018), whereas sand temperature adjacent to the nest did not. Development rates decline at these higher and suboptimum temperatures (Singh et al., 2020), meaning that these comparatively hot nests might be expected to take longer to hatch than nests that are 1–2°C cooler. Despite this, observed incubation temperatures of modelled nest 3 suggested hatching started around day 45 (Fig. S4).

Our modelled hatch times were based on a development rate function fitted to data from laboratory experiments on flatback turtle embryos at constant and fluctuating temperatures (Gammon et al., 2020), and this function is yet to be tested on natural nests. Similar functions for green and loggerhead turtles significantly overpredict incubation periods in natural nests (Booth et al., 2022). Several explanations have been proposed for this mismatch, including inaccuracies in model assumptions at high incubation temperatures when empirical data are lacking. Future work should prioritise collecting development rate data from natural nests experiencing thermal extremes and could draw on recent methods to pinpoint hatch times in underground nests (i.e. with accelerometers or a ‘hatching detector’; Booth et al., 2022; Rollinson et al., 2019).

### A generalised finite element model to predict embryonic development

While we modelled a typical flatback turtle nest, our broader aim was to present a universal approach that could be applied to other sea turtle species. Importantly, we used generalised parameters to model each nest and could obtain relatively accurate predictions. Our generalised parameters included egg chamber geometry (based on measurements for a different stock of flatback turtles; Koch et al., 2007), the arrangement of eggs (determined via a simulation), and the material properties of beach sand and eggs. We also applied a standardised heat flux to each egg based on the average temperature for each nest, and on a published relationship between incubation temperature, development stage and metabolic rates for flatback embryos (Gammon et al., 2021). In reality, embryonic heat fluxes would vary spatially owing to thermal gradients within the nest, but these differences likely have little impact on model accuracy. Our generalised method is ideal for testing on other species, as the necessary data on embryonic physiology are also available for loggerhead (*Caretta caretta*) and green turtle (*Chelonia mydas*) embryos (Ackerman, 1980; Booth and Astill, 2001a,b; Reid et al., 2009; Stubbs and Mitchell, 2018). Yet, we also acknowledge that thermal reaction norms for sex determination and development rates vary between different populations of the same species (Bentley et al., 2020b; Santidrián Tomillo, 2024) and so researchers should be cautious in applying physiological parameters from a different population to make predictions for a population whose embryonic physiology has not been studied. Fortunately, other species-specific parameters that we have incorporated in our model, such as nest depth and clutch size, are easier to measure.

### Model assumptions and limitations

Heat fluxes were assigned based on two different assumptions, resulting in two sets of finite element models: one assuming all eggs developed to hatching, and another adjusted to reflect the observed mortality of eggs, which included an entire clutch (nest 2). Although observed hatching success was low (38%), comparisons between these two sets of models showed that accounting for embryonic mortality had little effect on overall nest thermal dynamics. There are a several potential explanations for this. First, despite low hatching success, most unhatched eggs contained visibly developed embryos, indicating the metabolic heat would have been produced until mid- to late-stage development (Miller et al., 2017). Elevated nest temperatures of 35–37°C prior to hatching (Fig. S4) probably contributed to late-stage mortality, as these exceed optimal thermal ranges for embryonic development in other flatback turtle stocks (Howard et al., 2014). Second, a relatively low heat flux was assigned to each egg, so the impact of removing the heat flux of non-viable embryos was small. The tendency of our models to under-predict nest temperatures towards the end of development, when metabolic rates are highest, further suggests that our heat fluxes underestimated actual metabolic heat. Third, dead embryos may still contribute some heat through residual metabolism (Gammon et al., 2020) and microbial decomposition (Valverde et al., 2010), complicating the thermal effects of mortality. These findings point to the need for a more sophisticated approach to calculate heat fluxes, such as those based on dynamic energy budget theory (i.e. dynamic energy budget models; Kooijman, 2010), which provide a mechanistic framework for calculating metabolic rates throughout development (Stubbs et al., 2019). Future work could apply these approaches to nests with higher hatching success to further assess model accuracy. Notably, this study was conducted during an atypically hot year that resulted in low hatching success (∼45%) relative to the long-term average of ∼70% at Thevenard Island (Western Australian Department of Biodiversity, Conservation and Attractions, pers. commun.).

For simplicity and reproducibility, the equation we used to model individual hatching success was derived from a large multispecies dataset of observed incubation temperatures and hatching success of whole sea turtle clutches (Laloë et al., 2017). It is therefore necessary to make some simplifying assumptions when interpreting results for individual embryos. For example, low estimated hatching success for an individual embryo (i.e. <30%) might indicate a high probability of mortality, whereas a higher value (i.e. >50%) may not necessarily result in mortality, but could indicate sublethal effects, including reduced crawling and swimming speeds of hatchlings (Booth and Astill, 2001a,b; Booth et al., 2004; Booth and Evans, 2011) or developmental abnormalities (Zimm et al., 2017). Notably, for nest 3, only 60% of the embryos had a predicted probability of survival greater than 40%. Assuming these embryos experienced thermal stress but survived, this aligns closely with the nest's observed hatching success of 66%. Although this method is imperfect, we demonstrate how incorporating metabolic heat increases the number of eggs predicted to be exposed to potentially lethal conditions. Improved understanding of how long an embryo's exposure to high temperatures needs to be before it becomes lethal, and the sensitivity of this response across developmental stages, will allow hatching success to be predicted in a more nuanced manner.

The principal of homogenisation was used to estimate the thermal conductivity and specific heat capacity of beach and nest sand. The physical theory for heat conduction in material has been well developed for more than a century. Developments in methods to measure heat capacity of unsaturated porous materials began in the 1950s (de Vries, 1952) and continued to the present (Kluitenberg et al., 1993; Bristow et al., 1995; Lu et al., 2007). The principal of homogenisation underpins all these approaches and models derived from them have typical errors of <1%. Errors in the thermal modelling of real systems may stem from mis-specifying structural properties of porous medium, such as spatial heterogeneity in solid material types (i.e. quartz vs carbonate), bulk density and organic matter inclusions. However, the principal of homogenisation has been shown to well represent the thermal properties of such material heterogeneities when properly accounted for in the model (e.g. Wang et al., 2024). The conditions of most sandy beaches where turtle nesting occurs would tend to disfavour significant heterogeneity in solid material type and bulk density at scales relevant to an individual nest. Nevertheless, this heterogeneity in material/thermal properties could also be measured in the field (e.g. Speakman et al., 1998).

### Next steps: towards a fully mechanistic model

Although we demonstrate that finite element analysis can simulate heat transfer within a sea turtle nest, several improvements to our framework can be made. Specifically, as described above, our approach applied a nest-specific heat flux to each egg, based on calculations derived from observed incubation temperatures. Removing this dependency on empirical data would require the model's two boundary conditions (currently derived from observed sand temperatures) to be numerically modelled. The mechanistic microclimate model NicheMapR (Kearney and Porter, 2019) provides an ideal method to model sand temperatures at any depth. NicheMapR calculates below-ground temperatures by integrating local climate data with site-specific properties such as slope, aspect and sand reflectance, and its ability to accurately model sand temperatures at sea turtle nesting beaches is well established (Bentley et al., 2020a; Fuentes and Porter, 2013; Gammon et al., 2024; Stubbs et al., 2014). Modelling sand temperatures would remove the need for *in situ* loggers, which can be expensive to deploy at scale and be lost or shift depth; as demonstrated in the present study where most surface loggers were lost. Metabolic heat, represented by a ‘heat flux’, could then be calculated following the method presented here, using a representative node in the centre of the clutch as a proxy for incubation temperature. A further innovation would be to derive the metabolic heat flux directly from a mechanistic model of animal development and energy partitioning, such as dynamic energy budget (DEB) models (Kooijman, 2010), which have recently been completed for all sea turtle species (Marn and Kooijman, 2022).

A fully mechanistic version of our finite element analysis would be capable of exploring how almost any scenario might influence developmental outcomes, including combinations of nest interventions and climate change (Patrício et al., 2021). Sea turtle nests are threatened by a range of anthropogenic pressures which reduce hatching success and alter the primary sex ratio and health of hatchlings (Lutcavage et al., 2017). Active interventions to mitigate these pressures include relocating nests to avoid inundation (Garcıía et al., 2003), nest shading (Esteban et al., 2018), nest watering (Hill et al., 2015) and manipulating the clutch size of reburied nests (i.e. ‘clutch splitting’) to reduce the metabolic heat load (Clarke et al., 2021). Despite this range of interventions, their wide-scale implementation is limited by the complexity of understanding fine scale drivers of nest microclimates (Patrício et al., 2021), and how any manipulations might alter developmental outcomes. The modelling framework presented here allows the influence of these interventions on individual incubation temperatures to be explored and ultimately enables robust estimation of developmental outcomes for all embryos within a nest. For example, nest relocation protocols could be optimised by modelling the influence of the depth and dimensions of artificial nests on hatching success and sex ratios. Similarly, changes to the thermal properties of beach sand, such as those brought about by artificial beach nourishment or the accumulation of microplastics (Carson et al., 2011; Duncan et al., 2018; Lutcavage et al., 2017; Shamblott et al., 2021), can also be examined provided that the material properties of the modified ‘beach’ subdomain (Fig. 1) can be measured.

### Conclusion

Small changes in incubation temperature can have a major impact on the hatching success of sea turtle nests, which is critical to understanding population viability. Our ability to accurately forecast these outcomes hinges on our ability to precisely model the incubation temperatures experienced by all embryos within a nest through time, and under novel forcings such as climate change. Using the finite element method, we have demonstrated a means to individually model unique incubation trajectories which can be achieved with generalised data. Our methodology and workflow, although preliminary, can ultimately be applied to other sea turtle species and to any species with underground nests where metabolic heating is significant (e.g. freshwater turtles; Massey et al., 2019). To fully utilise spatially explicit estimates of incubation temperature, future research should seek to understand the mechanistic drivers of low hatching success in sea turtle embryos at high temperatures, which remain poorly understood (Patrício et al., 2021). Understanding these drivers will improve our ability to predict the impact of climate change on hatching success in sea turtles, ultimately informing effective conservation strategies.

## Supplementary Material

10.1242/jexbio.250238_sup1 Supplementary information
