# Supplementary material for: An application of finite element analysis predicts unique temperatures and fates for flatback sea turtle embryos
Source: J Exp Biol. 2025 Dec 4;228(23):jeb250238. doi: 10.1242/jeb.250238 (PMC12745931; doi:10.1242/jeb.250238)
Supplement: Supplementary information [file jexbio-228-250238-s1.pdf]

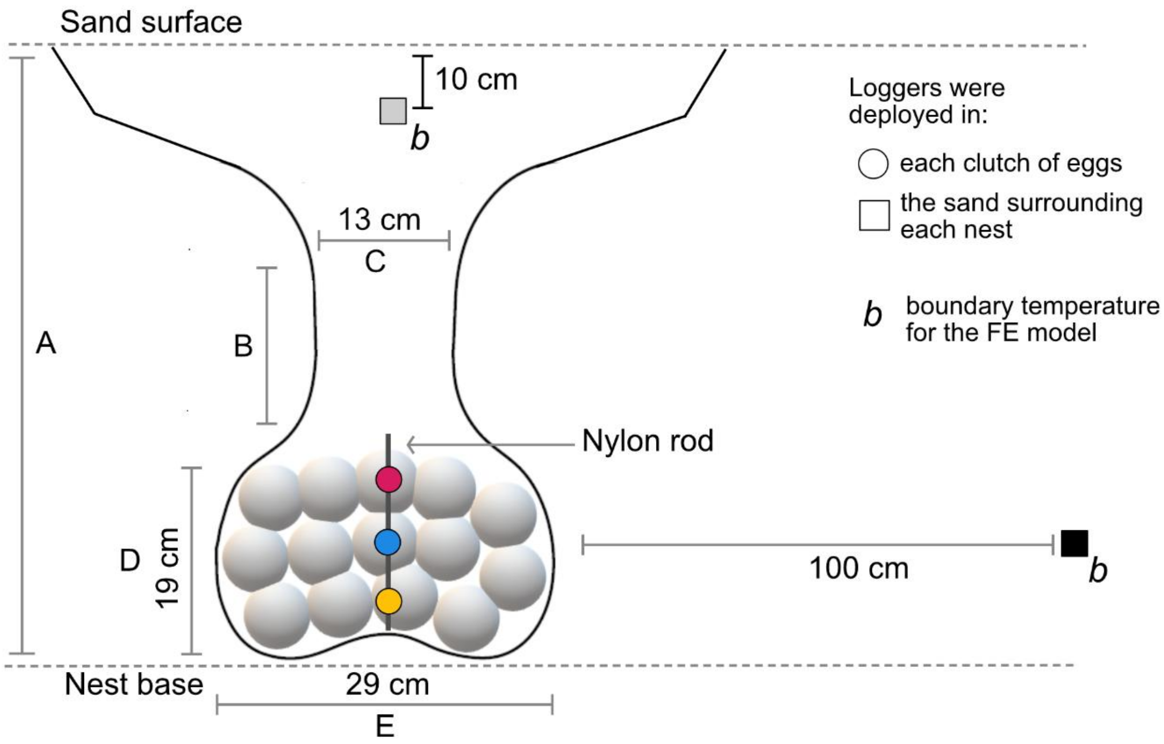

Fig. S1. Cross-section of the general geometry of flatback turtle nests (adapted from Koch et al., 2007), showing the placement of temperature loggers within the clutch (circles), and in the surrounding sand (squares). General dimensions of the geometry of flatback turtle nests (Koch et al. 2007) (C – E) were used to build a three-dimensional model of a nest chamber (C – E). Once established, this 'general' model of a flatback nest was customised to reflect the depth (A) of each modelled nest by adjusting the length of the nest neck (B).

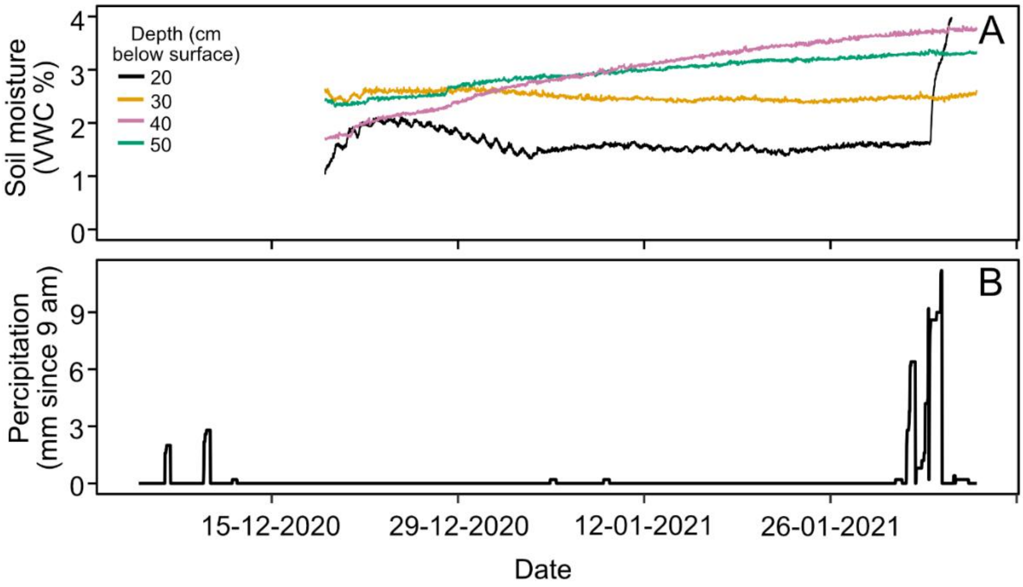

Fig. S2. Sand moisture (A) and rainfall (B) over the study period. Rainfall data were retrieved from the Australian Bureau of Meteorology weather station on Thevenard Island (station number 5084). Sand moisture was stable over the study period due to minimal rainfall, but rainfall in early February translated to a noticeable increase in the moisture of surface sand.

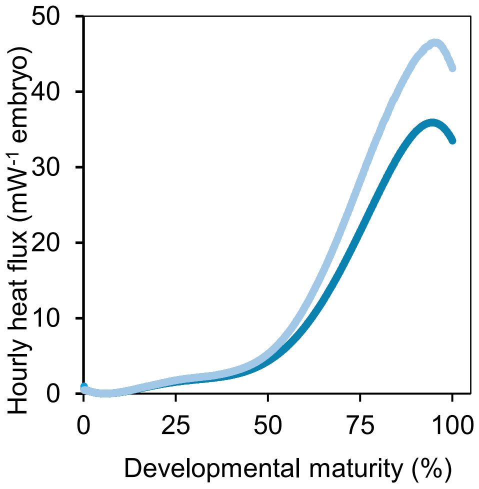

Fig. S3. Estimated metabolic heat produced by a single flatback turtle embryo developing at 29.5 °C (dark blue line), compared to metabolic heat adjusted to the hourly average incubation temperature recorded in nest 3 (light blue line). Hourly temperatures were averaged from three loggers placed at different locations within the clutch (see Fig. S1).

Journal of Experimental Biology • Supplementary information

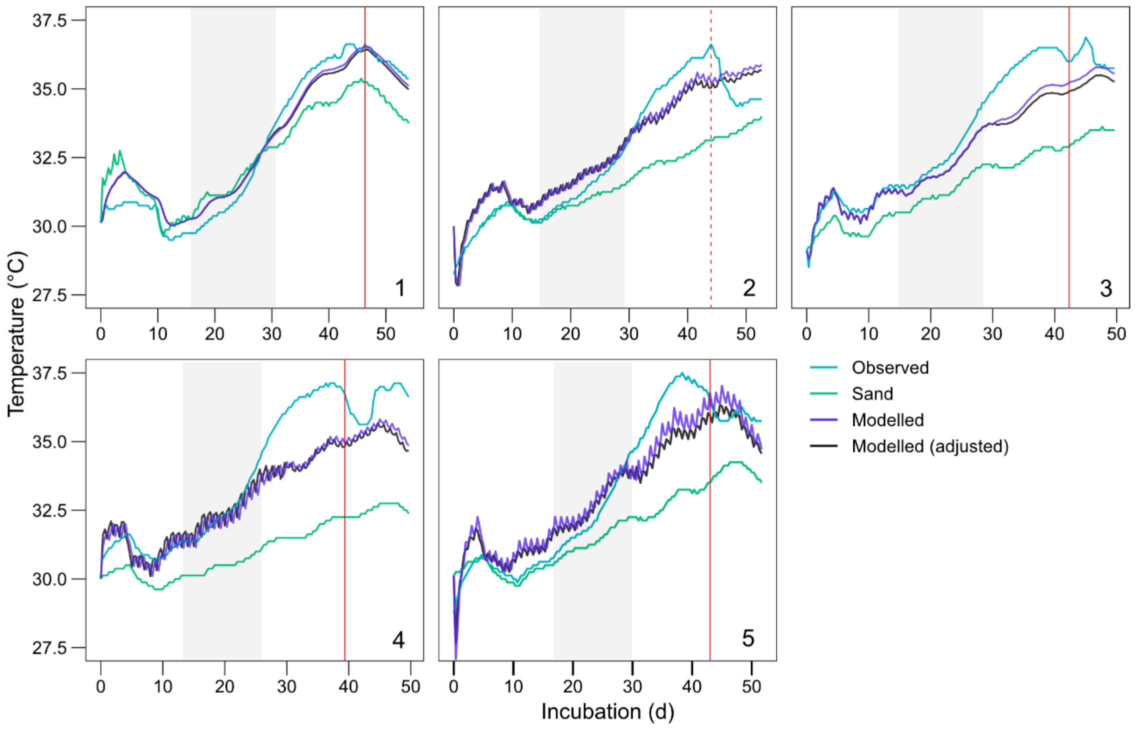

Fig. S4. Comparison of methods to predict the incubation temperature of central eggs in a clutch of flatback turtle eggs. Data are presented for five nests and shown as either measured incubation temperatures at the clutch centre (observed), the temperature of sand 1 m adjacent to the nest chamber (sand) at the same depth as the clutch centre, or an energy balance model underpinned by finite element analysis (modelled). Results for the energy balance model adjusted to reflect known mortality are also shown (modelled – adjusted). Daily amplitudes are detectable in modelled temperatures for nests 2, 4 and 5 due to diel cycles in boundary conditions at the sand surface. Earliest hatching (solid red vertical lines) is estimated based on a drop in observed incubation temperature, which typically occurs independently of surrounding sand temperatures. In these cases, late-stage mortality and rainfall near the expected hatching period (see Fig. S2) obscures the declines and makes the timing of emergence more difficult to identify. The thermosensitive period (TSP, grey shading) falls during the middle third of development from laying to hatching. No eggs hatched in nest 2, but 84% died very close to hatching (i.e., 'late-stage mortality). The TSP for this nest is based on a theoretical hatching date (dashed vertical line) that aligned with the time the mortality occurred.

Journal of Experimental Biology • Supplementary information

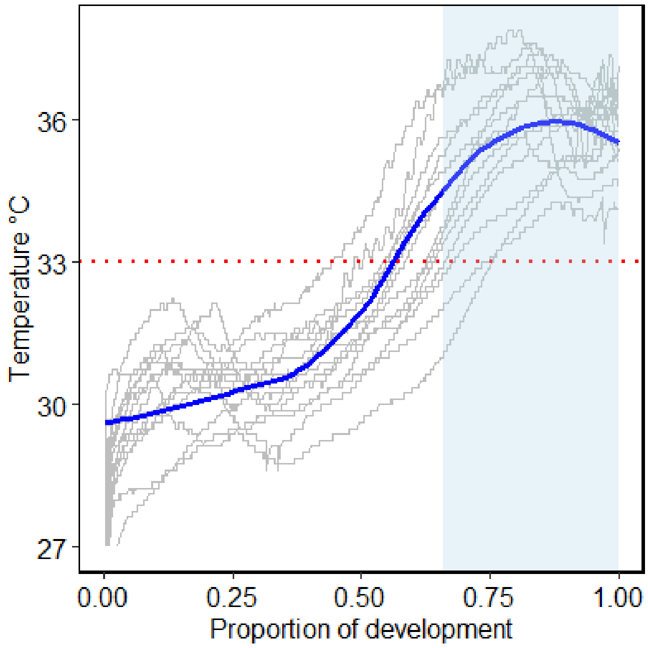

Fig. S5. Observed hourly incubation temperature at the centre of flatback turtle nests. Temperature of each nest (grey lines, *n* = 14) are plotted against the proportion of development (x-axis, from oviposition through to hatching), with the blue line showing the average development. Among those eggs which did not hatch, most were estimated to have died during the final trimester (blue shading) when nest temperatures exceeded 33 °C (red dotted line).

Journal of Experimental Biology • Supplementary information

Table S1. The density and moisture properties of beach sand from Thevenard Island, Western Australia.

Group	Bulk density (kg^{-1} m^3) ± SE	Moisture (% mass) ± SE
Surface sand (0 – 6.5 cm)	1384.70 ± 48.60	0.4 ± .3
Beach sand (30 – 36.5 cm)	1265.35 ± 66.05	4.0 ± .9
Beach sand (50 – 56.5 cm)	1299.12 ± 80.29	4.0 ± .8
Nest-chamber sand	1026.64[1]	4.0 ± .8[2]

1. Calculated for one nest, all other parameters were calculated from four nests.
2. Sand within the nest chamber is assumed to have the same moisture as beach sand.

Table S2. Hatching success parameters of modelled nests and heat flux scenarios for adjusted models.

Nest	1	2	3	4	5
Clutch size	38	43	51	59	61
Eggs with no development (%)[1]	5.3	2.3	3.9	8.5	3.3
Dead pre-term embryos (%)[2]	31.6	14.0	29.4	33.9	47.5
Dead full-term embryos (%)[3]	0.0	83.7	0.0	10.2	34.4
Hatch success (%)[3]	63.2	0.0	66.7	42.4	14.8
Pipped (but dead in-egg) (%)[3]	0.0	0.0	0.0	5.1	0

1. Eggs receiving no heat flux in mortality-adjusted models.
2. Eggs with heat flux until 67% of development in mortality-adjusted models.
3. Eggs with heat flux continuing through to hatching in mortality-adjusted models.

Journal of Experimental Biology • Supplementary information

Table S3. The thermal properties of beach sand

Material	Thermal conductivity (Wm^{-1} °C $^{-1}$)	Specific heat capacity (J kg^{-1} °C $^{-1}$)	Density (Kg^{-1} m^3)	Reference
Sand (dry, quartzite)	6.5	741	2650	Zeller & Pohl (1971)
Water	0.6	4177.6	1000	Mounanga et al. (2004)
Air	0.025	1.225	1.225	Mounanga et al. (2004)

Table S4. Volume fractions (ϕ) of the material components of beach sand and clutch sand.

Material	ϕ_{water}	ϕ_{air}	ϕ_{sand}
Beach sand	.0513	.516	.433
Clutch sand	.0410	.613	.346

Table S5. The composition of eggs and hatchlings in sea turtles and other reptile species.

Stage	Percent water[1]	Species	Reference
Egg	78.8%	Flatback turtle (*N. depressus*)	Hewavisenthi & Parmenter (2001)
Hatchling	77.5% (73.9% yolk-free)	Flatback turtle (*N. depressus*)	Hewavisenthi & Parmenter (2001)
**Stage**	**Density[2] (Kg^{-1} m^3)**	**Species**	**Reference**
Egg (albumen)	1020	Freshwater crocodile (*Crocodylus johnstoni*) Saltwater crocodile (*Crocodylus porosus*)	Webb et al. (1987)
Egg (yolk)	1040 – 1060	Freshwater crocodile (*C. johnstoni*) Saltwater crocodile (*C. porosus*)	Webb et al. (1987)
Subembryonic fluid	1000	Freshwater crocodile (*C. johnstoni*) Saltwater crocodile (*C. porosus*)	Webb et al. (1987)
Adult	1040	Leatherback turtle (*D. coriacea*)	Fossette et al. (2010)

1. Calculated as the change in mass between fresh and dried samples.
2. Densities are within 6% of the density of water (1000 Kg^{-1} m^3; Mounanga et al. 2004).

Journal of Experimental Biology • Supplementary information

Table S6. Average difference (Δ) between temperatures at the base and centre of the clutch for observed and modelled data[1] for nests with different clutch sizes.

Nest[2]	Clutch size	Δ observed (°C)	Δ modelled (°C)
1	38	0.2 ± 0.0	0.1 ± 0.0
2	43	0.5 ± 0.0	0.2 ± 0.0
3	51	0.9 ± 0.0	0.2 ± 0.0
4	59	1.1 ± 0.0	0.3 ± 0.0
5	61	0.8 ± 0.0	0.3 ± 0.0
	**Average Δ**	**0.7 ± 0.0**	**0.2 ± 0.0**

1. Models were not adjusted to reflect embryo mortality
2. Numbers refer to the nests in Table 1, and their corresponding nest-specific characteristics.

Equations relating to the material properties of sand and other modelled sub-domains

Equation S1

Bulk density (kg^{-1} m^3) of sand cores:

$$\frac{\text{Dry soil weight (g)}}{\text{Soil volume (cm}^3\text{)}} \times 1000$$

Equation S2

Percent water saturation (by mass) of sand cores:

$$\frac{(\text{Wet weight (g)-dry weight (g)})}{\text{dry weight (g)}} \times 100$$

Equation S3

Mori-Tanaka's equation of homogenisation:

$$\lambda_c = \frac{\sum_{i=s,\,w,\,a} \phi_i \lambda_i \chi_i}{\sum_{i=s,\,w,\,a} \phi_i \chi_i} \quad \text{where } \chi_i = \frac{3\lambda_M}{2\lambda_M + \lambda_i}$$

where,

λ_c = thermal conductivity of the homogenised material (i.e. beach sand); W m^{-1} °C^{-1}

s, w, a = materials (sand, water, air)

ϕ_i = volume fraction of the ith material (sand, water, or air)

λ_i = thermal conductivity of the ith material (sand, water, or air); W m^{-1} °C^{-1}

M = the matrix (i.e. sand)

Journal of Experimental Biology • Supplementary information

Equation S4

The rule of mixtures (Laloui & Loria, 2020):

$$\overline{X}_{C_p} = \sum_{i=s,\, w,a} \phi_i X_i$$

where,

$\bar{X}_{C_p}$ = specific heat capacity of homogenised material; J kg^{-1} °C^{-1}

s, w, a = materials (sand, water, or air)

ϕ = volume fraction of the ith material (sand, water, or air)

χ_i = specific heat capacity of the ith material (sand, water, or air); J kg^{-1} °C^{-1}

Equations relating to the estimation of embryonic heat fluxes

Equation S5

Development rate (r_a; % per day) is expressed as a function of temperature *(T):*

$$r_a = b_1 10^{-v^2(1-b_5+ b_5 v^2)}$$

where

$$u = \frac{(T - b_3)}{(b_3 - b_2)} - c_1,$$

$$v = \frac{u + e^{b_4}}{c_2},$$

$$c_1 = \frac{1}{(1 + 0.28b_4 + 0.72ln(1 + b_4))},$$

$$c_2 = 1 + \frac{b_4}{(1 + 1.5b_4 + 0.39b_4^2)}.$$

Parameters were fitted to data from flatback turtle embryos from the same genetic stock as the present study (Gammon et al., 2021), where maximum development rate (*b_1*) was 2.5% per day, occurring at a corresponding temperature (*b_3*) of 34.7 °C. Refer Gammon et al., (2021) for fuller details.

Journal of Experimental Biology • Supplementary information

Equation S6

An hourly heat flux was estimated for each embryo (mW $embryo^{-1}$) as a function of developmental maturity (i.e., 0 – 100%, with 100% denoting hatching stage), using a fifth order polynomial regression fitted to data on flatback embryos incubated at 29.5 °C:

$$-7.8x10^{-8}d^{5} + 1.6x10^{-5}d^{4} + -1.0x10^{-3}d^{3} + 2.9x10^{-2}d^{2} + 0.65$$

Where d is the cumulative percentage of development. Note that metabolic heat was estimated by converting metabolic rates ($\dot{V}CO_2$ and $\dot{V}O_2$) to their energy equivalence (see Gammon et al., 2021 for a full description).

Equation S7

Hourly Q_{10} temperature coefficients were calculated using development rates derived from the non-linear development rate function (Equation S5), following Van Hoft's (1844) equation:

$$Q_{10} = \left(\frac{R_2}{R_1}\right)^{10/(T_2-T_1)}$$

where,

R_1 = 1.9 and T_1 = 29.5 °C, and

R_2 = development rate (% per day, calculated using Eq S5) at the corresponding incubation temperature (T_2) for that hour of incubation.

Journal of Experimental Biology • Supplementary information

Equation S8

Hourly metabolic rates (mW^{-1} embryo) were estimated for each embryo as a function of its developmental maturity, and observed temperatures using the equation:

$$R_2 = R_1 \, Q_{10}^{(T_2 - T_1)/10\,°\mathrm{C}}$$

Where,

R_1 is the hourly metabolic rate based on developmental maturity (Eq S6), at a reference temperature of 29.5 °C (T_1),

T_2 is the hourly incubation temperature (°C), and

Q_{10} is the temperature co-efficient based on development rates at T_1 and T_2 (Eq S7).

Supplementary references

Fossette, S., Gleiss, A. C., Myers, A. E., Garner, S., Liebsch, N., Whitney, N. M., Hays, G. C., Wilson, R. P., & Lutcavage, M. E. (2010). Behaviour and buoyancy regulation in the deepest-diving reptile: the leatherback turtle. *Journal of Experimental Biology, 213*(Pt 23), 4074-4083. doi:10.1242/jeb.048207.

Gammon, M., Bentley, B., Fossette, S., & Mitchell, N. (2021). Metabolic rates and thermal thresholds of embryonic flatback turtles (*Natator depressus*) from the north west shelf of Australia. *Physiological and Biochemical Zoology, 94*(6), 429-442. doi:10.1086/716848.

Hewavisenthi, S., & Parmenter, C. J. (2001). Influence of incubation environment in the development of the flatback turtle (*Natator depressus*). *Copeia,* 2001(3), 668-682. Doi:10.1643/0045-8511.

Koch, A. U., Guinea, M. L., & Whiting, S. D. (2007). Effects of sand erosion and current harvest practices on incubation of the flatback sea turtle (*Natator depressus*). *Australian Journal of Zoology, 55*(2), 97-105.

Laloui, L. & Loria, A. F. R. (2020). Chapter 3: Heat and mass transfers in the context of energy geostructures. In *Analysis and design of energy geostructures: theoretical essentials and practical application, 69 – 135.*

Lighton, J. R., Bartholomew, G. A., & Feener, D. H. (1987). Energetics of locomotion and load carriage and a model of the energy cost of foraging in the leaf-cutting ant Atta colombica Guer. *Physiological Zoology*, *60*(5), 524-537.

Mechtly E. 1964. The international system of units: physicalconstants and conversion factors. Scientific and TechnicalInformation Division, National Aeronautics and Space Administration, Washington, DC

Mounanga, P., Khelidj, A., & Bastian, G. (2004). Experimental study and modelling approaches for the thermal conductivity evolution of hydrating cement paste. *Advances in Cement Research, 16*(3), 95-103.

Van't Hoff, J. H. (1884). 'Etudes de Dynamique Chimique.' (Muller: Netherlands).

Webb, G. J. W., Manolis, S. C., Whitehead, P. J., & Dempsey, K. (1987). The possible relationship between embryo orientation opaque banding and the dehydration of albumen in crocodile eggs. *Copeia, 1987*(1), 252-257. doi:10.2307/1446070.

Zeller, R., & Pohl, R. (1971). Thermal conductivity and specific heat capacity of noncrystalline solids. *Physical Review B,* 4(6), 2029.

Journal of Experimental Biology • Supplementary information
